# Human papillomavirus (HPV) in young women in Britain: Population-based evidence of the effectiveness of the bivalent immunisation programme and burden of quadrivalent and 9-valent vaccine types

**DOI:** 10.1016/j.pvr.2017.01.001

**Published:** 2017-01-06

**Authors:** Clare Tanton, David Mesher, Simon Beddows, Kate Soldan, Soazig Clifton, Kavita Panwar, Nigel Field, Catherine H. Mercer, Anne M. Johnson, Pam Sonnenberg

**Affiliations:** a Research Department of Infection & Population Health, University College London, Mortimer Market Centre, London WC1E 6JB, UK; b Centre for Infectious Disease Surveillance and Control (CIDSC), Public Health England, 61 Colindale Avenue, London NW9 5EQ, UK; c Virus Reference Department, Public Health England, 61 Colindale Avenue, London NW9 5EQ, UK; d NatCen Social Research, 35 Northampton Square, London EC1V 0AX, UK

**Keywords:** Probability sample survey, HPV, Prevalence, Women, Immunisation programme, HPV vaccine

## Abstract

**Background:**

In 2008, the UK introduced an HPV immunisation programme in girls. Population-based prevalence estimates of bivalent (HPV-16/18), quadrivalent (HPV-6/11/16/18) and 9-valent (HPV-6/11/16/18/31/33/45/52/58) vaccine types, and comparison over time, are needed to monitor impact, evaluate effectiveness and guide decision-making on vaccination strategies.

**Methods:**

The third National Survey of Sexual Attitudes and Lifestyles (Natsal-3) in 2010-12, tested urine for HPV from 2569 sexually-experienced women aged 16–44. We report type-specific HPV prevalence and compare results with 1798 women in Natsal-2 (1999–2001) using age-adjusted prevalence ratios (APR).

**Findings:**

In Natsal-3, 4.2% of women aged 16-44y were positive for HPV‐16/18 and 2.9% for HPV-6/11. In 16–20 year olds, 4.5%, 10.8% and 20.7% had at least one bivalent, quadrivalent or 9-valent vaccine type, respectively. Three-dose vaccine coverage was 52.0% in women aged 18-20y. In this age group, HPV-16/18 prevalence was lower in Natsal-3 than Natsal-2 (5.8% vs 11.2%; APR=0.48[95%CI: 0.24–0.93]), however, prevalences of HPV-6/11, HPV-31/33/45 and HPV-52/58 were unchanged. HPV-16/18 prevalence was also unchanged in women aged 21-44y (APR=0.85[0.61–1.19]).

**Interpretation:**

These probability surveys provide evidence of the impact of the bivalent immunisation programme. Reductions were specific to HPV-16/18 and to the age group eligible for vaccination. However, substantial vaccine-preventable HPV remains.

## 1 Introduction

Over 99% of cervical cancer is caused by persistent infection with certain high-risk (HR) types of human papillomavirus (HPV) [1], [2]. In 2008, the UK introduced an HPV immunisation programme using the bivalent vaccine that protects against HPV-16 and HPV-18, which cause over 70% of cervical cancers [3]. This school-based programme offers the HPV vaccine to all girls aged 12–13 years. A catch-up programme was implemented in schools and general practice over the first few years for girls aged up to 18 years. In 2012, the programme switched to the quadrivalent vaccine, which additionally protects against HPV-6/11, which cause 90% of genital warts [4]. Clinical trials have shown very high vaccine efficacy against the two high-risk types included in both vaccines [5], [6] as well as some evidence for cross-protection against HPV-31 for both vaccines and HPV-33 and HPV-45 for the bivalent vaccine [7], [8]. More recently, phase III trials have demonstrated the efficacy of a new 9-valent vaccine, which, in addition to the types in the quadrivalent vaccine, directly protects against HPV types 31/33/45/52/58 [9], responsible for a further 20% of cervical cancers [10].

Population-based prevalence estimates of bivalent, quadrivalent and 9-valent vaccine types, and comparison over time, are needed to monitor impact and guide decision-making on vaccination strategies. It is likely that the cost-effectiveness of using the 9-valent vaccine will be considered by the Joint Committee on Vaccination and Immunisation (JCVI) in the UK and other policy-making bodies internationally. The National Surveys of Sexual Attitudes and Lifestyles (Natsal) are large probability sample surveys of the British population that include demographic and behavioural information linked to STI test results. Results from Natsal-2, using data collected in 1999–2000, provided baseline population estimates of type-specific HPV (from urine samples) in women aged 18–44 years prior to the introduction of the bivalent immunisation programme [11]. Using data from Natsal-3, conducted a decade later using the same methodology, we have previously reported age-specific prevalences of HR-HPV [12]. We have also shown a reduction in HPV-16/18 in women aged 18–20 years following the introduction of the immunisation programme [12], with vaccine coverage of 62% in those eligible for the catch-up campaign [13].

In this paper, we extend the analysis of Natsal-3 data to include estimates of a wide range of HPV types, including HPV-6/11, HPV-31/33/45 and HPV-52/58. In addition, more detailed comparison of results from the two surveys is used to evaluate bivalent vaccine effectiveness. These include effects on HPV-16/18 prevalence and non-vaccine types in the age groups targeted by the catch-up campaign, as well as examining changes in sexual behaviour in the decade between the surveys.

## 2 Material and methods

### 2.1 Participants and procedure

Natsal-3 was a probability sample survey of 15,162 men and women (8869 women) aged 16–74 years and resident in Britain. Interviews were carried out during 2010–2012. Participants were interviewed using computer-assisted personal interviewing, with computer-assisted self-interview for the more sensitive questions. Women eligible for the national HPV immunisation programme (those born on or after 1 September 1990, up to 21 years by the end of the interview period) were asked “Have you ever been vaccinated against cervical cancer (received HPV vaccine)?”. Further methodological details have been described elsewhere [14], [15]. The Natsal-3 fieldwork dates, and the age of participants (youngest age 16) meant that some of the participants were eligible for vaccination, the vast majority of whom would have been vaccinated as part of the catch-up programme.

At the end of the interview, a sample of participants aged 16–44 years who reported at least one lifetime sexual partner were invited to provide a urine sample to be tested for STIs, including HPV, and 60% agreed (similar agreement in men and women) [12]. Written consent was provided for testing without return of results [16]. Details of the urine collection, processing and testing procedures have been described previously [12], [14]. Samples were collected using the FirstBurst urine collection device [17] and were tested for HPV using an in-house Luminex-based genotyping assay [18]. HPV types were classified as high-risk using the IARC Monograph Working Group classification [19].

The previous Natsal survey (Natsal-2) was carried out in 1999–2001 [20] and interviewed 11,161 men and women (6399 women) aged 16–44 years. Sample selection and procedures were similar to Natsal-3. Urine samples were requested from a sample of 18–44 year olds, of whom 71% agreed to provide a sample [21] with HPV results available for 1851 women [11]. Samples were collected using a urine collection cup. HPV testing protocols for Natsal-2 samples were identical to those used for Natsal-3.

### 2.2 Statistical analysis

Data were analysed using Stata (v13.1), accounting for the stratification, clustering and weighting of the sample. To account for differences in the probability of selection for and response to providing a urine sample, an additional weight, derived from a logistic regression model, was applied to the urine data for analysis, as described previously [12], [14].

We report prevalence (and 95% confidence intervals (CIs)) of key HPV type combinations and HPV types in women by age group (16–20, 21–24 and 25–44). Data for comparison between Natsal-2 and Natsal-3 were available for women aged 18–44. We compare prevalence of HPV-16/18, HPV-31/33/45, HPV-52/58 and HPV-6/11 in the age group eligible for vaccination (aged 18–20 years) and the prevalence of HPV-16/18 in older women (aged 21–44 years). We present age-adjusted prevalence ratios (APR), calculated using generalised linear models with a log link function, for the association between survey and these key type combinations.

Changes in sexual behaviour in men and women aged 18–44, in the decade between Natsal-2 and Natsal-3, have been previously reported in detail [15]. To explore whether changes in HPV prevalence could be due to changes in sexual behaviour, we compared a number of markers of sexual behaviour in Natsal-2 and Natsal-3, in sexually-experienced women aged 18–20 years, the age group eligible for vaccination.

### 2.3 Ethics

Natsal-3 was granted ethical approval by the Oxfordshire Research Ethics Committee A (Reference: 09/H0604/27). Natsal-2 obtained ethical approval from University College Hospital, North Thames Multicentre, and all local research ethics committees in Britain.

## 3 Results

### 3.1 Prevalence of HPV types

In Natsal-3, HPV results were available for 2569 sexually-experienced women aged 16–44 years. The prevalence of HPV types and type combinations, stratified by age, is shown in Table 1. For all types, the prevalence was higher in the younger women (aged 16–20 and 21–24 years) than in those aged 25 or over.

**Table 1:** Type-specific prevalence of HPV in women aged 16–44 years.

	**16–20 years**		**21–24 years**		**25–44 years**		**All**	
	%	95%CI	%	95%CI	%	95%CI	%	95%CI
*Denominator (unwt., wt)*	*493, 279*		*464, 294*		*1612, 1616*		*2569, 2189*	
**HPV type combinations**								
High-risk types^a^	26.7%	[22.6–31.2]	25.0%	[21.0–29.5]	12.4%	[10.7–14.3]	15.9%	[14.4–17.5]
Types 31/33/45	5.6%	[3.6–8.7]	6.4%	[4.4–9.3]	2.2%	[1.6–3.0]	3.2%	[2.6–3.9]
Types 31/33/45/52/58	12.7%	[9.7–16.4]	10.8%	[8.1–14.2]	5.0%	[4.0–6.2]	6.8%	[5.8–7.8]
Types 6/11	7.0%	[4.9–9.8]	5.1%	[3.1–8.4]	1.8%	[1.3–2.6]	2.9%	[2.3–3.7]
**HPV types**								
HPV-6	2.7%	[1.4–5.1]	3.1%	[1.5–6.5]	0.6%	[0.3–1.1]	1.2%	[0.8–1.8]
HPV-11	5.1%	[3.3–7.7]	2.0%	[1.1–3.6]	1.5%	[1.0–2.2]	2.0%	[1.5–2.6]
HPV-16	3.5%	[2.3–5.4]	5.8%	[3.9–8.4]	3.0%	[2.2–4.1]	3.5%	[2.8–4.3]
HPV-18	1.0%	[0.5–2.2]	1.6%	[0.8–3.5]	0.6%	[0.3–1.1]	0.8%	[0.5–1.2]
HPV-26	0.6%	[0.2–1.8]	0.1%	[<0.1–1.0]	0.4%	[0.2–0.9]	0.4%	[0.2–0.8]
HPV-31	1.7%	[0.6–4.6]	2.1%	[1.1–3.9]	0.7%	[0.4–1.1]	1.0%	[0.7–1.5]
HPV-33	1.7%	[0.7–4.1]	1.2%	[0.5–2.6]	0.3%	[0.1–0.8]	0.6%	[0.4–1.0]
HPV-39	4.7%	[3.0–7.3]	2.7%	[1.5–4.9]	0.9%	[0.6–1.4]	1.6%	[1.2–2.2]
HPV-45	3.4%	[2.0–5.8]	4.0%	[2.4–6.7]	1.4%	[1.0–2.1]	2.0%	[1.6–2.6]
HPV-51	3.4%	[2.1–5.4]	2.3%	[1.3–4.0]	1.3%	[0.8–2.1]	1.7%	[1.2–2.3]
HPV-52	5.4%	[3.7–7.9]	2.8%	[1.5–5.2]	1.8%	[1.2–2.7]	2.4%	[1.9–3.1]
HPV-53	4.8%	[3.1–7.3]	5.6%	[3.7–8.4]	2.2%	[1.4–3.2]	2.9%	[2.3–3.8]
HPV-56	4.9%	[3.3–7.3]	3.6%	[2.1–6.2]	1.0%	[0.6–1.7]	1.8%	[1.4–2.5]
HPV-58	3.2%	[2.0–5.2]	2.8%	[1.5–5.2]	1.3%	[0.8–2.0]	1.7%	[1.2–2.3]
HPV-59	3.1%	[1.8–5.3]	2.2%	[1.2–4.2]	0.8%	[0.5–1.3]	1.3%	[0.9–1.7]
HPV-66	1.8%	[0.9–3.5]	1.2%	[0.5–2.8]	1.2%	[0.7–2.1]	1.3%	[0.9–1.9]
HPV-68	1.7%	[0.9–3.3]	2.2%	[1.1–4.3]	1.4%	[0.8–2.2]	1.5%	[1.0–2.2]
HPV-70	0.7%	[0.3–1.6]	1.4%	[0.7–2.7]	1.2%	[0.8–1.8]	1.1%	[0.8–1.6]
HPV-73	3.0%	[1.9–4.9]	1.7%	[0.7–4.0]	0.7%	[0.4–1.2]	1.1%	[0.8–1.6]
HPV-82	0.5%	[<0.1–2.2]	<0.1%	[<0.1–0.7]	<0.1%	[<0.1–0.5]	0.1%	[<0.1–0.4]

a Defined as Group 1 (16, 18, 31, 33, 35, 39, 45, 51, 52, 56, 58, 59) & Group 2 A (68) HPV types [19].

In total, 4.2% of 16–44 year olds were positive for bivalent vaccine types (HPV-16/18) (Table 2). Prevalence was 4.5% in 16–20 year olds and 7.4% in 21–24 year olds. Vaccine coverage with three doses in these age groups was 57.2% (52.1–62.1) and 5.6% (3.5–8.7), respectively. HPV-16 was the most common type overall (prevalence of 3.5%), but not in 16–20 year olds. Although the prevalence of HPV-6/11 was only 2.9% overall, there was a strong association with younger age (prevalence in those aged 16–20 was 7.0%).

**Table 2:** Prevalence of HPV vaccine types in women aged 16–44 years.

	**16–20 years**		**21–24 years**		**25–44 years**		**All**	
	%	95%CI	%	95%CI	%	95%CI	%	95%CI
*Denominator (unwt., wt)*	*493, 279*		*464, 294*		*1612, 1616*		*2569, 2189*	
**Vaccine HPV types**								
Bivalent types (HPV-16/18)^a^	4.5%	[3.0–6.6]	7.4%	[5.3–10.3]	3.6%	[2.7–4.8]	4.2%	[3.4–5.2]
Quadrivalent types (HPV-6/11/16/18)	10.8%	[8.3–14.0]	12.2%	[9.2–16.1]	5.3%	[4.3–6.7]	7.0%	[6.0–8.1]
9-valent types	20.7%	[17.0–24.9]	20.6%	[16.8–25.0]	9.9%	[8.4–11.6]	12.7%	[11.3–14.2]

a Only one woman was positive for both HPV-16 and HPV-18.

Prevalence of other HR-HPV types found in the 9-valent vaccine was high – 6.8% overall (Table 1). Table 2 shows that one in eight women aged 16–44 and one in five women aged <25 years, were positive for at least one 9-valent vaccine type. In 16–20 year olds, 4.5%, 10.8% and 20.7% had at least one bivalent, quadrivalent or 9-valent vaccine type, respectively.

### 3.2 Markers of early vaccine effectiveness

Table 3 shows the prevalence of key combinations of HPV types in women aged 18–20 years in Natsal-2 and Natsal-3 in women. In this age group, in Natsal-3, 52.0% (46.1–57.9) of women reported having received all three doses of the HPV vaccine, with an additional 6.0% (3.6–9.8) reporting having received one or two doses.

**Table 3:** Prevalence of HPV among women aged 18–20 years in Natsal-2 and Natsal-3.

	**Natsal-2**		**Natsal-3**			
	%	95%CI	%	95%CI	APR	95%CI
*Denominator (unwt., wt)*	*140, 150*		*331, 199*			
HPV-16/18	11.2%	[6.7–18.3]	5.8%	[3.9–8.6]	0.48	[0.24–0.93]
HPV-31/33/45/52/58	12.9%	[7.9–20.3]	14.8%	[11.1–19.6]	1.19	[0.69–2.05]
HPV-31/33/45	5.0%	[2.4–10.0]	7.2%	[4.5–11.4]	1.50	[0.66–3.44]
HPV-52/58	9.5%	[5.3–16.3]	8.7%	[6.1–12.3]	0.94	[0.48–1.80]
HPV-6/11	9.5%	[5.0–17.5]	8.9%	[6.1–12.7]	0.91	[0.44–1.89]

APR – age-adjusted prevalence ratio for Natsal-3 vs. Natsal-2

**Fig. 1:**
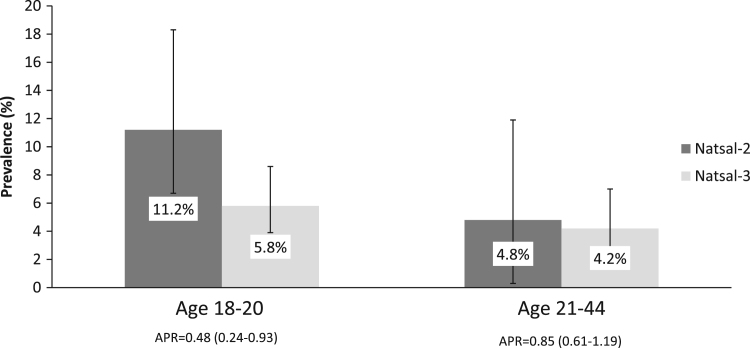
Prevalence of HPV-16/18 among women aged 18–20 years and 21–44 years in Natsal-2 and Natsal-3.

In women aged 18–20 years, the prevalence of HPV-16/18 was lower in Natsal-3 than Natsal-2 (5.8% vs. 11.2%; APR=0.48 (0.24–0.93)), whereas for older women (aged 21–44 years, Fig. 1), no significant difference in HPV-16/18 prevalence was seen between the two surveys. In Natsal-3, the prevalence of HPV-16/18 was lower in women aged 18–20 years who had been vaccinated compared to unvaccinated women (OR 0.39 (95%CI 0.17–0.91) p=0.030; AOR (adjusted for age and number of lifetime partners) 0.46 (95% CI 0.20–1.05) p=0.065).

In the women aged 18–20 years, the prevalence of any one or more of the HR-HPV types found in the 9-valent vaccine (31/33/45/52/58) was similar in the two surveys, as was the prevalence of HPV-6/11. There was also no evidence for a difference in HPV-31/33/45 prevalence between Natsal-2 and Natsal-3.

### 3.3 Changes in sexual behaviour

Between Natsal-2 and Natsal-3, the proportion of sexually-experienced 18–20 year old women reporting first heterosexual intercourse before the age of 16 increased from 29.2% (24.5–34.4) to 38.8% (34.5–43.3) (p=0.005). However, there was little evidence for a change in other markers of risky sexual behaviour. For example, the number of women reporting ≥5 lifetime partners remained unchanged (39.7% (33.8–46.0) vs. 43.6% (38.9–48.3) (p=0.332)) as did the number reporting ≥1 new partner in the past year (55.0% (49.1–60.8) vs 57.7% (53.2–62.0), p=0.483).

## 4 Discussion

Population-based HPV prevalence estimates, and comparison of prevalence over time, are needed to inform decisions about future policy for HPV vaccination in the UK. This paper presents prevalence estimates for a range of HPV types circulating in the British population in 2010–2012, and shows that there remains a significant amount of vaccine-preventable HPV. Even amongst 16–20 years who were eligible for the immunisation programme, 5%, 11% and 21% of women were infected with at least one of the bivalent, quadrivalent or 9-valent vaccine types, respectively. The Natsal data do nevertheless provide an early indication of population-based effectiveness of the bivalent vaccine in women in the age groups eligible for vaccination, with a ~50% reduction seen in the prevalence of HPV-16/18 in 18–20 year olds (APR=0.48 comparing Natsal-3 to Natsal-2). This reduction was specific to the bivalent vaccine types and to the age group eligible for the catch-up campaign. A recent meta-analysis showed that, in countries with female vaccination coverage of at least 50%, HPV-16/18 has reduced by 68% [22].

The strength of Natsal is that the HPV data are collected together with detailed information on sexual behaviour. This allows us to examine the counterfactual – could HPV have reduced in this age group as a result of reductions in risky sexual behaviour in the decade between the surveys? We have previously reported that there has been little change in sexual risk behaviour in those aged 18–44 between the two surveys [14], [15]. In the younger age groups, who would have been eligible for vaccination, we found that the proportion of women reporting first sex before age 16 had increased, although there was no change in other key risk behaviours suggesting that the prevalence of HPV would have remained stable or increased in a scenario without vaccination. National surveillance data from the UK also suggests that there has been a reduction in the prevalence of the bivalent vaccine HPV types in the population [23], [24]. As shown in a recent meta-analysis [22], with longer duration of the programme [22], we may see a reduction in HPV in older women, in men, and in HPV-31/33/45 due to cohort effects, herd immunity and cross-protection.

Two other UK studies have considered the population-level impact of a national HPV vaccination programme on HPV prevalence. One considered changes in HPV type-specific prevalence in a high-risk population of young women in England attending for chlamydia screening [23]. This study demonstrated a reduction in the HPV vaccine types following the introduction of the bivalent vaccine from 17.6% pre-vaccination (2008) to 8.5% 2–3 years post-vaccination (2010-11) and 4.0% 4–5 years post-vaccination (2012-13) in the 16–18 year old age-group with highest vaccination coverage. In Scotland, among 20–21 year old women attending for cervical screening, the prevalence of HPV-16/18 reduced from 28.8% in 2009 to 10.1% in 2013 (3-dose vaccination coverage 72.7%) [24]. Our data show little evidence of a change in the prevalence of HPV-31/33/45 or HPV-31/33/45/52/58 among 18–20 year olds (APR=1.50 and 1.19 respectively, comparing Natsal-3 to Natsal-2). Conversely, the Scottish data showed a reduction in HPV-31/33/45 from 13.0% in 2009 to 6.3% in 2013, despite an increase in prevalence of other HR-HPV types over this time period. It is also encouraging that a decrease in cervical abnormalities (CIN2 and CIN3) has been seen in Scotland following the introduction of the national programme using the bivalent vaccine [25].

The two Natsal studies are large population-based probability sample surveys, conducted using similar methods a decade apart, which provide an opportunity to assess the impact of public health interventions on a national scale. The combination of obtaining detailed demographic and behavioural data, linked to biological specimens, provides both subjective (eg reported vaccine coverage) and objective (eg HPV results) measures of process and outcome. Each of the Natsal datasets is weighted to account for survey participation and differential urine selection probabilities and response [14]. However, the findings need to be considered in the context of the potential limitations. Firstly, we have tested urine samples for type-specific HPV infection. Whilst urine samples are generally easier to collect and more acceptable than genital samples, it is has been shown that urine specimens have a lower sensitivity to detect HR-HPV and HPV-16/18 infection compared to genital swabs [18]. This lower sensitivity suggests that the prevalences reported would be an underestimate of the true HPV type specific prevalences in the population. Using urine in consecutive surveys means that the results can be used to compare relative changes in type-specific HPV infection. Secondly, although the overall sample size for Natsal is large, the study was not powered to assess prevalence estimates, especially in subgroups, such as by both age and gender, and for the rarer HPV types, or to make comparisons between surveys. Despite this, we were able to show significant reductions in HPV-16/18 between the surveys. Finally, the data on vaccine coverage is based on self-report and may be subject to bias. Comparison of the proportion of respondents who reported having been vaccinated (62% in that age group and time period in Natsal-3), with nationally reported data [24], [26], [27], [28], [29], [30]), suggests that there is unlikely to be significant misclassification.

Whilst we show reductions in the bivalent HPV vaccine types, there remains a considerable HPV burden in the population. The 9-valent HPV vaccine has been licensed for use in Europe (3-dose schedule) [31]. If this vaccine were to be considered for the national vaccination programme then these data will provide a useful baseline to compare population-based changes in the HPV prevalence of these types. However, the types included in the 9-valent vaccine are less common in cervical disease and invasive cancer than the bivalent vaccine types [3]. Policy-makers, such as JCVI, need to consider these factors, as part of a comprehensive comparison of the available vaccines, including measurement of cost-effectiveness.

These data compare HPV prevalence in the British population before and after the introduction of a national HPV vaccination programme and strengthen evidence of a population effect against the HPV types included in the vaccines. Of note, is that these are the first population-based results in the UK general population, rather than in specific groups such as those attending for chlamydia screening [23] or cervical screening [24], [25]. The majority of women eligible to receive the HPV vaccine in this survey will have been vaccinated as part of the catch-up vaccination programme. Detailed description of vaccine coverage from Natsal-3, including by age, school year and number of doses, and analysis of demographic and behavioural factors associated with not being vaccinated, have been previously reported [13]. Those at higher risk, based on sexual behaviour, were less likely to be vaccinated.

These largely baseline data contribute to the evaluation of the effectiveness and cost-effectiveness of the current strategy and future decisions on who should be vaccinated, vaccine choice and schedule. Future surveys will include a larger proportion of vaccinated women, vaccinated at a younger age, and should see further reductions in the HPV vaccine types and allow further consideration of changes in the closely-related HPV types. Longer-term expectations, given higher coverage with the quadrivalent vaccine, or the introduction of the 9-valent vaccine, would be a reduction in cervical cancer precursors, cervical cancer and genital warts [32].

## Funding

The study was supported by grants from the Medical Research Council [Grant no. G0701757] and the Wellcome Trust [Grant no. 084840], with contributions from the Economic and Social Research Council and Department of Health. NF is supported by a National Institute for Health Research Academic Clinical Lectureship. SC was funded to undertake independent research supported by the National Institute for Health Research (NIHR Research Methods Programme, Fellowships and Internships, NIHR-RMFI-2014-05-28). The views expressed in this publication are those of the author(s) and not necessarily those of the NHS, the National Institute for Health Research or the Department of Health. The sponsors had no role in the study design; in the collection, analysis and interpretation of data; in the writing of the report; and in the decision to submit the article for publication.

## Conflicts of Interest

A.M.J. has been a Governor of the Wellcome Trust since 2011.
